# Calcification of Blood-Vessels Following Injury

**Published:** 1887-04

**Authors:** W. F. Westmoreland

**Affiliations:** Atlanta, Ga.


### Clinic Reports.

### CALCIFICATION OF BLOOD-VESSELS FOLLOWING INJURY.

REPORT OF SURGICAL CLINIC, ATLANTA POLYCLINIC, MARCH 2D, 1887, SERVICE OF W. F. WESTMORELAND, JR., M. D.

The case I present now is suffering from an injury received thirty-seven years ago. The patient is sixty-one years old. He says that while engaged in splitting logs thirty-seven years ago a heavy piece of timber fell from considerable distance and struck his ankle, crushing it, so that for a time he was unable to use it at all. After he recovered sufficiently, he resumed work, as he says, with a sore still on the front part of the leg, extending to the foot. In this condition he worked for several years, when he sustained another injury similar to the first, which kept him in bed for eight months. He says he has never been able to walk since this last injury without crutches.

His condition now calls for amputation. You will notice that the foot is more than twice the size of the uninjured one, while the entire anterior portion of the leg from about the middle to near the instep is one large ulcer. There is perfect anchylosis of the ankle-joint, with extreme contraction of the anterior muscles of the leg, flexing the foot on the leg to the extent that the toes point almost directly upwards.

To attempt to treat this by any other means than amputation would be folly. We will therefore place the patient under the influence of ether and amputate the leg at the upper third.

Before making the amputation the leg is shaved and thoroughly washed with a nail-brush and soap ; then with a solution of bichloride of mercury 1 to 1,000. Now Esmarch's bandage is applied, beginning as you see at the toes and carried above the knee, when we apply the tourniquet and the bandage is removed. We now protect the seat of operation by towels dipped in the solution of corrosive sublimate. We make the flap by the circular method, splitting it up on the outer side to leave a place for the drainage tube. The bone is now divided and we are ready for the ligatures. We will use in this case, as we do in all others where we can, catgut ligature, and will attempt to ligate the vessels before removing the tourniquet, so as to avoid any unnecessary hemorrhage.

We find the vessels have undergone calcification, and it will be a difficult matter to get the ligature to hold on account of the brittle condition of the vessels due to this calcification. You see that it is necessary to use great caution in tying them; whenever any great pressure is brought to bear on them they break. The arteries have all been secured and the tourniquet is now removed. You will observe that there is free venous hemorrhage; it will be necessary to ligate the veins in order to arrest this hemorrhage. One of these veins is so brittle that it is impossible to get a ligature to hold, and we will endeavor to arrest the hemorrhage in it by packing the vein with catgut. You will notice that I am packing it firmly, filling the calibre with the catgut by means of a probe, and now there seems to be no more bleeding.

We will thoroughly wash the wound with bichloride mercury solution 1 to 2,000 and defer the final dressing for sometime until we are satisfied there will be no further hemorrhage. We close the wound first with a continuous suture of large catgut deeply taken, then with a continuous suture of small catgut we bring the edges of the wound into direct apposition.

We now insert a large rubber drainage tube.

We first put on several thick layers of bichloride gauze and fasten it with gauze bandages, then several layers of sublimated wood-wool and secure it with bandages. Then a thick layer of absorbent cotton and secure it. We will now apply a well-padded posterior splint extending from the upper part of the thigh to below the stump and bandage it on firmly so as to insure perfect rest.

We will remove the drainage tubes on the seventh day and will be able to send patient home well in two weeks.

[Fifteen days after the operation the wound had entirely healed and patient sent to his home in the country. There was only one dressing applied.—REPORTER.]

